# Identification of a Three-RNA Binding Proteins (RBPs) Signature Predicting Prognosis for Breast Cancer

**DOI:** 10.3389/fonc.2021.663556

**Published:** 2021-07-12

**Authors:** Yang Liu, Hefen Sun, Xuan Li, Qiqi Liu, Yuanyuan Zhao, Liangdong Li, Baojin Xu, Yifeng Hou, Wei Jin

**Affiliations:** ^1^ Department of Breast Surgery, Key Laboratory of Breast Cancer in Shanghai, Fudan University Shanghai Cancer Center, Shanghai, China; ^2^ Department of Oncology, Shanghai Medical College, Fudan University, Shanghai, China

**Keywords:** breast cancer, RNA-binding protein, prognostic model, bioinformatics analysis, MRPL12, Mrpl13, POP1

## Abstract

**Background:**

To date, breast cancer remains the primary cause of tumor-related death among women, even though some leap-type developments of oncology have been done to slash the mortality. Considering the tumor heterogeneity and individual variation, the more reliable biomarkers are required to be identified for supporting the development of precision medicine in breast cancer.

**Methods:**

Based on the TCGA-BRCA and METABRIC databases, the differently expressed RNA binding proteins (RBPs) between tumor and normal tissues were investigated. In this study, we focused on the communal differently expressed RBPs in four subtypes of breast cancer. Lasso-penalized Cox analysis, Stepwise-multivariate Cox analysis and Kaplan–Meier survival curve were performed to identify the hub RBP-coding genes in predicting prognosis of breast cancer, and a prognostic model was established. The efficiency of this model was further validated in other independent GSE20685, GSE4922 and FUSCC-TNBC cohorts by calculating the risk score and performing survival analysis, ROC and nomogram. Moreover, pathologic functions of the candidate RBPs in breast cancer were explored using some routine experiments *in vitro*, and the potential compounds targeting these RBPs were predicted by reviewing the Comparative Toxicogenomics Database.

**Results:**

Here, we identified 62 RBPs which were differently expressed between the tumor and normal tissues. Thereinto, three RBPs (MRPL12, MRPL13 and POP1) acted as independent risk factors, and their expression pattern also correlated with poor prognosis of patients. A prognostic model, built with these 3-RBPs, possessed statistical significance to predict the survival probability of patients with breast cancer. Furthermore, experimental validations showed that down-regulating the expression of endogenous MRPL12, MRPL13 or POP1 could dramatically suppress the cellular viability and migration of breast cancer cells *in vitro*. Besides, some compounds (such as the Acetaminophen, Urethane and Tunicamycin) were predicted for curing breast cancer *via* targeting MRPL12, MRPL13 and POP1 simultaneously.

**Conclusion:**

This study identified and established a 3-RBPs-based signature and nomogram for predicting the survival probability of patients with breast cancer. MRPL12, MRPL13 and POP1 might act as oncogenes in maintaining cellular viability and accelerating metastasis of breast cancer cells, implying the possibility of which to be designed as biomarkers and/or therapeutic targets for breast cancer.

## Introduction

Breast cancer is an extraordinarily heterogeneous set of neoplasms with sophisticated pathogenesis and alarming incidence, especially being a formidable threat to women. Only in 2018, almost 2.1 million of breast cancer were diagnosed worldwide, accounting for 24.2% of all the new cancer cases in females (1). Although advances in the early diagnosis and clinical intervention slashed the mortality of breast cancer significantly, breast cancer remains a feared, refractory and primary cause of tumor-related death among women. Following a curative intent while avoiding being overtreated, the novel personalized and precision medicine that constructed upon the set of reliable tumor biomarkers and targets are emerging and ongoing. Therefore, the more reliable and available tumor biomarkers would be identified, the more eligible and prescriptible therapeutic strategies would be adopted in clinical treatment of breast cancer.

RNA-binding proteins (RBPs) are ubiquitously expressed in cells and play important roles in regulating the cellular physiological and pathological processes (2). Conventionally, RBPs participate in the formation of ribonucleoprotein complexes *via* binding to the modular sequence and/or structural motifs of target RNAs, and then dynamically alter the fate or function of RNAs, such as regulating its stability, localization, modification and translation (3–5). Moreover, some proteins that are lacking the canonical RNA-binding domains also moonlight as RBPs under specific cellular context (6). For instance, the 3-hydroxyacyl-CoA dehydrogenase type 2 (HSD17B10) not only behaves as a mitochondrial enzyme which is involved in the oxidation of isoleucine and branched-chain fatty acids, but also binds to the 5’-end of most mitochondrial tRNAs that recruits RNase P to regulate the stability and activity of target mitochondrial tRNAs (7–9).

Accumulating evidence revealed an inextricable causality between the dysregulated RBPs and various human diseases, including malignant tumor (10). Intrinsically, throughout the tumorigenesis and progression, RBPs act as participants and/or coordinators to regulate the proliferation, apoptosis, senescence, angiogenesis, metastasis and invasion of cancer cells (11, 12). For instance, aberrant over-expressed IGF2BP1 in hepatocarcinoma could accelerate cell proliferation *via* motivating the expression of its target mRNAs, including *c-Myc*, *Ki67* and *β-TrCP1* (13). DND1 might competitively interact with *miR-221* against *BIM* and stabilized *BIM* mRNA, thus haploinsufficiency of DND1 assisted the breast cancer cells in escaping apoptosis *via* decreasing the expression of *BIM* (14). Moreover, ESRP1/2 might contribute to the epithelial–mesenchymal transition of cancer cells by regulating the alternative splicing of *FGFR2* and *CD44*, and in turn accelerated metastasis (15, 16).

Given that RBPs are critical regulators and effectors of cancer, they also should be regarded as hallmarks and potential targets for early diagnosis, prognosis and clinical intervention of cancer. NOP14/MRPS23/POP1 exhibit prognostic value in colorectal cancer, while APOBEC3C/DCAF13/EIF4E3/EZR possess significance in predicting prognosis of breast cancer (17, 18). Moreover, the Ribavirin, an antiviral guanosine analog, is able to slacken tumorigenesis by competitively interacting with the 5’-Cap of *eIF4E* mRNA, subsequently impeding the translation and transposition of eIF4E-regualted *Cyclin D1* (19). Nevertheless, the physiological functions and pathological features of most RBPs remain unknown, especially for the unconventional RBPs.

The benefit of integrating the high-throughput genomic analysis of clinical specimens and relative epidemiological research, systematic studies of RBPs in one specific type or subtype of cancer will be more helpful to identify the exclusive biomarkers and potential therapeutic targets in one restrictive type of cancer, such as the ER-signaling pathway has been applied as the major therapeutic target in Luminal-like breast cancer, but is inefficient in basal-like breast cancer. Therefore, in this study, the molecular classification of breast cancer was employed as a precondition to identify the extra biomarkers for breast cancer. A series of stepwise bioinformatics analysis were carried out in five transcriptional profile datasets (TCGA-BRCA, METABRIC, FUSCC-TNBC, GSE20685 and GSE4922), and identified a number of RBPs which might be involved in breast cancer. Furthermore, *in vitro* experimental validation indicated that some of these candidate RBPs would be used as generic prognostic biomarkers and potential therapeutic targets for breast cancer.

## Materials and Methods

### Acquisition of Data Sources and Identification of Differently Expressed RBPs

The RNA-sequencing information of 1,001 cases of breast cancer samples and 113 cases of normal breast tissue samples with clinicopathological information were obtained from the TCGA-BRCA database (http://portal.gdc.cancer.gov/), wherein 653 cases of Luminal A, 150 cases of Luminal B, 39 cases of HER2-riched as well as 159 cases of TNBC samples were included. In this study, we referred to the human RBP catalog reported by Gerstberger et al. (2), which consisted of 1,542 RBPs but only 1,538 RBP-coding genes are recorded and available in the TCGA-BRCA database (**Supplementary Table 1**). To identify the differently expressed RBP-coding genes between the breast tumor and normal tissues, the “edgeR”, “pheatmap” and “ggplot2” packages were employed and performed in each molecular subtype with normal tissues as control, in view of |log_2_ (fold change)| ≥1 and FDR <0.05. Then, the “VennDiagram” package was used to identify the communal differently expressed RBP-coding genes between the four subtypes of breast cancer. Moreover, in order to increase the credibility of bioinformatic prediction, other datasets were introduced with detailed RNA-sequencing data and complete clinicopathological information. The METABRIC dataset (n = 1m893) were obtained from cBioPortal (http://www.cbioportal.org/), the GSE20685 cohort (n = 327) and the GSE4922 cohort (n = 249), as well as our local FUSCC-TNBC dataset (GSE118527) which contains 360 cases of TNBC samples and 88 cases of paired para-carcinoma samples were obtained from GEO (http://www.ncbi.nlm.nih.gov/). Data from TCGA-BRCA and METABRIC databases were normalized using the “edgeR” package, while data from GEO databases were normalized using the “limma” package.

### Construction of Protein–Protein Interaction (PPI) and Pathways Interaction Network

The STRING database (*version 11.0*, http://www.string-db.org/) was used to evaluate the protein–protein interaction information and biological functions of all the differently expressed RBPs. The PPI network was visualized by *Cytoscape 3.8.0*, and the interaction modes of biological processes along with molecular functions were built by ClueGO in *Cytoscape*.

### Identification of Hub Genes

In the TCGA-BRCA dataset, Lasso-penalized Cox regression analysis and stepwise multivariate Cox regression analysis were conducted in turn to screen hub genes from the above differently expressed RBPs, that might be prognostic signature for overall survival (OS) and/or disease-free survival (DFS) in breast cancer patients. Then, the candidate genes derived from Cox regression analysis were submitted to assess their prognostic significance for survival of patients, who were grouped by the Youden index of candidate mRNA expression pattern into high- or low-expression set. Only if the candidate is significantly in both the TCGA-BRCA and METABRIC datasets, would be considered as a hub gene for further study.

### Establishment and Validation of the Prognostic Model

A prognostic model for breast cancer was built based on the signature of these hub genes predicted as above, which was constructed by a linear combination of the Cox regression coefficients (β) multiplied with its mRNA expression pattern. In brief, the risk score of each patient was calculated by the formula that risk score = (β_Gene1_ × expression_Gene1_) + (β_Gene2_ × expression_Gene2_) +…+(β_GeneN_ × expression_GeneN_). Patients were grouped into high- or low-risk set according to the Youden index of their risk score, and the survival difference between these two sets was analyzed by the Kaplan–Meier estimation combined with a log-rank test in the testing datasets (TCGA-BRCA and METABRIC). Moreover, the predictive value of this model was further applied and validated into other datasets, such as the public GSE20685 cohort, GSE4922 cohort and our local FUSCC-TNBC dataset (GSE118527).

### Independent Prognostic Analysis

To investigate whether this prognostic model was independent of other clinical parameters, such as age, ER status, PR status, HER2 status and TMN stage, univariate and multivariate analyses were carried out in the TCGA-BRCA dataset using Cox regression model method with forwarding stepwise procedure where *p <*0.05 was considered as statistically significant.

### Construction and Validation of a Predictive Nomogram

For application of the prognostic model, a predictive nomogram was constructed using all the independent prognostic factors derived from the multivariate Cox regression analysis. Discrimination of the nomogram was assessed by the concordance index (C-index) using a bootstrap method with 1,000 resamples, and prediction probabilities of the nomogram against actual survival of patients were evaluated by plotting the calibration curve.

### Acquisition and Processing of Samples

For experimental validation, altogether 88 cases of tumor tissues and paired para-carcinoma tissues were obtained from patients with TNBC, who had undergone radical resection surgical of tumor between 2007 and 2014 with a median duration of follow-up of 45.8 months at Fudan University Shanghai Cancer Center, China. The dissected samples were stored in liquid nitrogen immediately until extraction of total RNA. Meanwhile, a series of immortalized breast cancer cell lines (such as MDA-MB-231, bone metastatic 231-BO, lung metastatic 231-HM), the mammary epithelial cell line MCF10A as well as its derived pre-malignant cell line MCF10^DCIS^, malignant cell line MCF10^Ca1h^ and MCF10^Ca1a^ were employed. According to the recommendations from the American Type Culture Collection (ATCC), the MDA-MB-231, 231-BO and 231-HM cells were routinely maintained in *Leibovitz’s L-15* basal medium supplemented with 10% fetal bovine serum and 1% penicillin–streptomycin, while the MCF-10A, MCF-10^DCIS^, MCF-10^Ca1h^ and MCF-10^Ca1a^ cells were grown in *DEME/F-12* medium supplemented with 5% Chelex-treated horse serum, 0.01 mg/ml human insulin, 20 ng/ml epidermal growth factor, 100 ng/ml cholera toxin and 500 ng/ml hydrocortisone.

### Realtime-qPCR Analysis

For evaluating the transcriptional expression pattern of the hub genes, the total RNA was isolated from tissues and cells using TRIzol reagent (*Invitrogen#15596026*), and cDNA was synthesized using the PrimeScript™ RT reagent kit (*TaKaRa#RR037*) under the guidance of the manufacturer’s instructions. Realtime-qPCR was performed using the TB Green^®^ Premix Ex Taq™ II kit (*TaKaRa#RR82LR*), and the sequence of primers were listed as below: MRPL12-F 5’-ATCCCCATAGCGAAAGAACGG-3’; MRPL12-R 5’-GGACGAGGTTGATGCCTTGG-3’; MRPL13-F 5’-ACATAAACCTGTGTACCATGCAC-3’; MRPL13-R 5’-GGTAGCCAGTATGCGAAGAGT-3’; POP1-F 5’-AGACAGCTGCTTCTGGAGGGT-3’; POP1-R 5’-TGACCCAGGCACCAGCAATTC-3’; Besides, GAPDH was used as internal control for normalization, whose sequence was designed as GAPDH-F 5’-GGAGCGAGATCCCTCCAAAAT-3 and GAPDH-R 5’-GGCTGTTGTCATACTTCTCATGG-3’.

### Down-Regulating the Expression of the Candidate Endogenous RBP-Coding Genes

For each candidate RBP, two shRNA oligos against different sites of CDS (coding sequence) were designed and synthesized (**Supplementary Table 2**). The pLKO.1-puro plasmids (*Addgene#8453*) carried the specific shRNA oligos for *MRPL12*, *MRPL13* or *POP1* were constructed, and be co-transfected into HEK-293T cells with the pVSVg (*Addgene#8454*) and psPAX2 (*Addgene#12260*) for packaging specific lentivirus. Then, the specific lentivirus was harvested and be co-cultured with target cells. Realtime-qPCR analysis was applied to assess the interference effect of these shRNAs on the expression of target genes.

### Cellular Migration and Viability Assay

For assessing the influence of candidate RBPs on the migration of breast cancer cells, transwell assay was performed. Cells in logarithmic growth phase were harvested and suspended in DMEM-H medium, and seeded into the upper chambers (0.8 μm, *BD#353097*) at 5 × 10^4^ cells/well. While the lower chambers were filled with 600 μl of DMEM-H medium supplemented with 20% FBS. After 14 h incubation, cells were fixed and stained with crystal violet staining solution, while the non-invading cells could be removed using swab. Then, the invaded cells were counted under microscope in six random fields at a magnification of 100×. Cloning efficiency was detected for evaluating the influence of these candidate RBPs on cellular viability. Cells in logarithmic growth phase were harvested and suspended in complete growth medium, and seeded into 6-well clusters at 5 × 10^2^ cells/well. About 14 d later, cell colonies were fixed and stained with crystal violet staining solution. Then, the colony-forming efficiency was calculated by comparing the number of colonies between the gene-edited cells and its parental cells.

### Target–Drug Interaction Network Analysis

For constructing a target–drug interaction network, the online database CTD (Comparative Toxicogenomics Database, http://ctdbase.org/) was introduced. By retrieving the CTD database, some potential compounds that might be involved in regulating these prognostic biomarkers were identified, and visualized by using *Cytoscape 3.8.0*.

### Statistical Analysis

*R v3.5.0* and *SPSS v23.0.0.0* were employed for statistical analysis, and the *GraphPad Prism v8.4.3* was used for scientific drawing. False discovery rate (FDR) ≤0.05 was designed as threshold for filtering the significantly difference between tumor and normal tissues in bioinformatics analysis, while a Student’s t-test was used for comparing the difference between the test and control groups in experimental validations. Meanwhile, Kaplan–Meier curves and Cox regression analysis with log-rank *p* test were applied into survival analysis, in which *p <*0.05 was considered as statistically significant.

## Results

### 62 RBP-Coding Genes Were Differently Expressed in Breast Tumor Specimens

**Figure 1:**
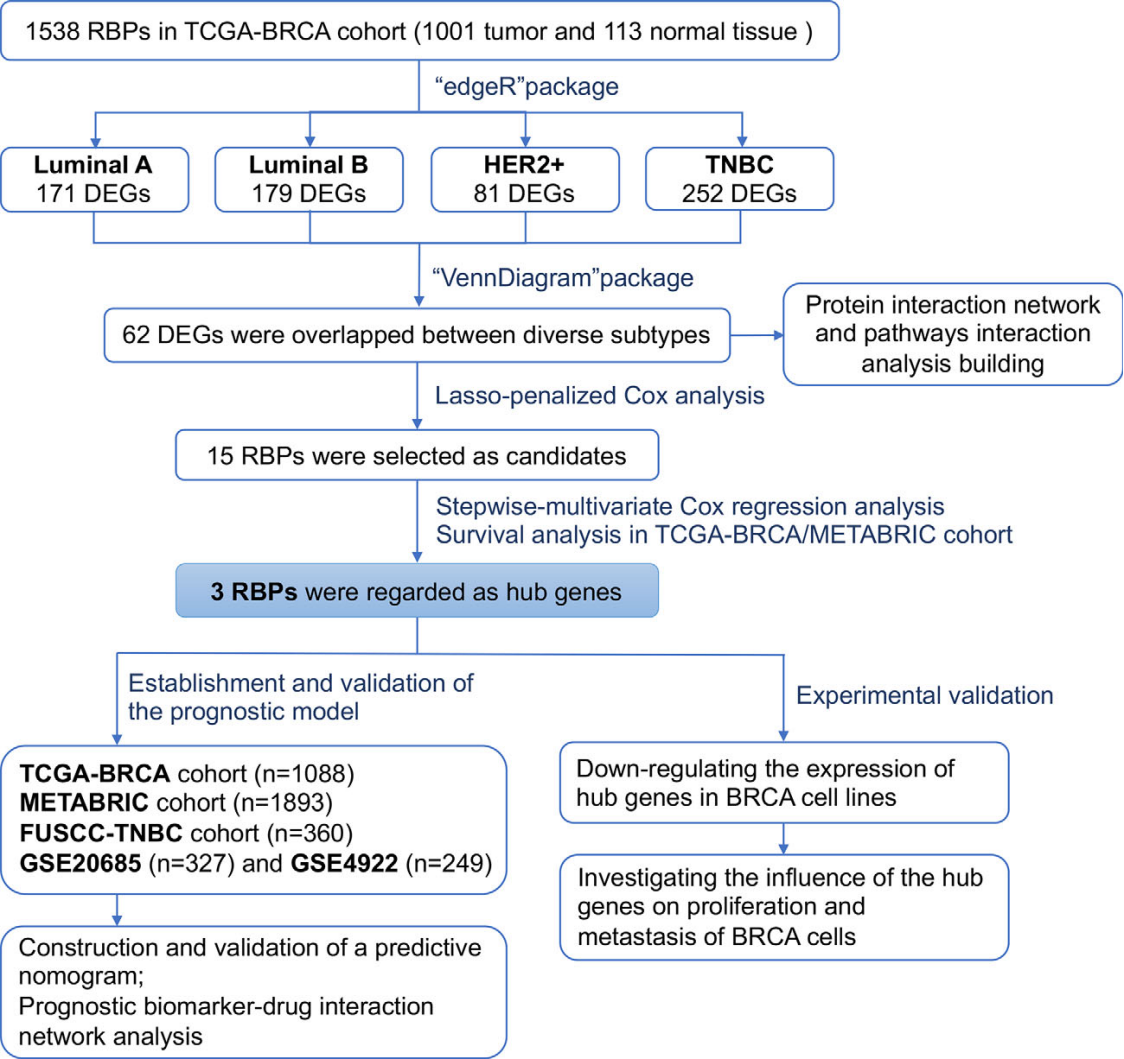
Flowchart showing the scheme of this study in identifying a RBPs signature predicting prognosis for breast cancer.

**Figure 2:**
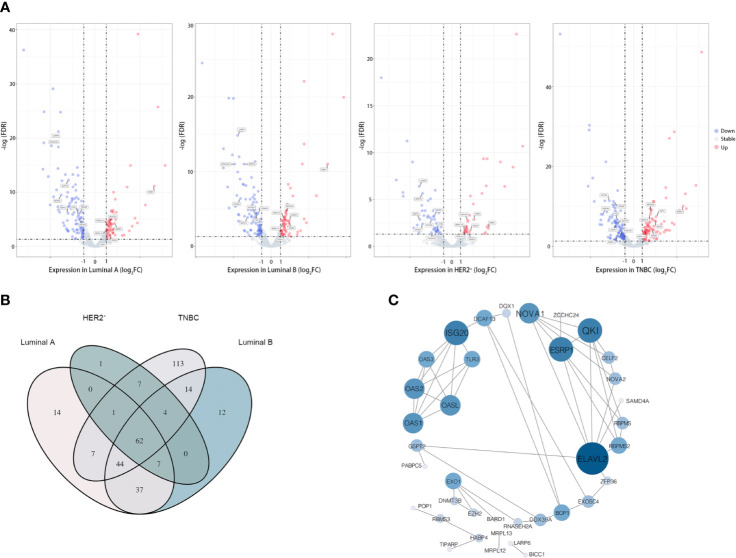
62 RBPs were differently expressed in breast tumor samples. **(A)** Volcano plot showing the differently expressed RBPs in diverse subtype of breast cancer, from left to right was the Luminal A subgroup, Luminal B subgroup, HER2-positive subgroup (HER2^+^) and Triple-negative subgroup (TNBC). Moreover, 15 candidate RBPs which were screened by Lasso-penalized Cox regression analysis were labeled; **(B)** “Venn Diagram” illustrated the communal differently expressed RBPs between subtypes of breast cancer; **(C)** Protein–protein interaction (PPI) network of the overlapped 62 candidate RPBs in breast cancer, size of the node representing the value of relationship between RBPs.

Based on the available RNA sequencing data from TCGA-BRCA database, we conducted a deep analysis of 1,538 RBP-coding genes and identified a total of 323 genes that be differently expressed between tumor and adjacent normal tissues (**Figure 1** and **Supplementary Tables 3**, **4**). Given that breast cancer is a heterogeneous and polygenic disease, which exhibits biological distinctness and behavioral difference, we further employed the recognized four molecular subtyping of breast cancer into analysis. Compared to normal tissues, there were 171 RBPs differently expressed in tumor tissues of the Luminal A subtype, 179 RBPs differently expressed in the Luminal B subtype, 81 RBPs differently expressed in the HER2-riched subtype while 252 RBPs differently expressed in TNBC samples (**Figure 2A** and **Supplementary Table 4**). Notably, 62 differently expressed RBPs were communal in four subtypes of breast cancer, wherein 24 RBPs were up-regulated but 38 RBPs were down-regulated in tumor tissues (**Figure 2B** and **Supplementary Table 4**).

### Functional and Pathway Interrelation Analysis of the Differently Expressed RBPs

**Figure 3:**
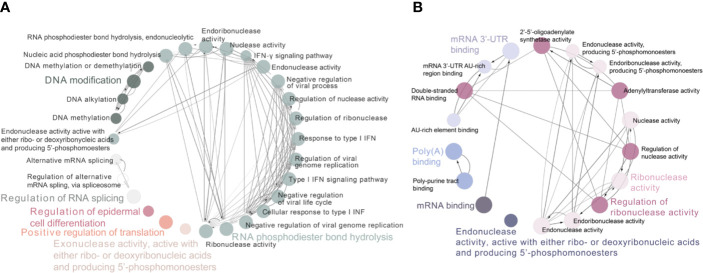
Functional and pathway interrelation analysis of the differently expressed RBPs. **(A)** Interrelation analysis between biological process; **(B)** Interrelation analysis between molecular functions pathways that be driven by these identified RBPs. Nodes in the same GO term had been highlighted in one color.

To determine the function of these differently expressed RBPs in oncogenesis or even deterioration of breast cancer, we constructed the protein–protein interaction (PPI) network and analyzed the pathways interrelation. According to the STRING 11.0 database, 62 PPI nodes and 56 edges with a *p*-value of 2E−12 were plot (**Figure 2C**). Notably, ELVAL2 showed the highest relationship with other candidate RBPs, but it had been winnowed out in further Cox regression analysis, it might be due to its negligible contribution to predict prognosis of breast cancer. For GO analysis, the biological process of these 62 RBPs was mainly enriched in RNA phosphodiester bond hydrolysis, DNA modification and regulation of RNA splicing, while these 62 RBPs were also played important roles in regulating the ribonuclease activity, mRNA 3’-UTR binding, mRNA binding as well as poly-A binding (**Figure 3**).

### Establishment and Validation of a 3-RBPs-Signature Based Prognostic Model

**Figure 4:**
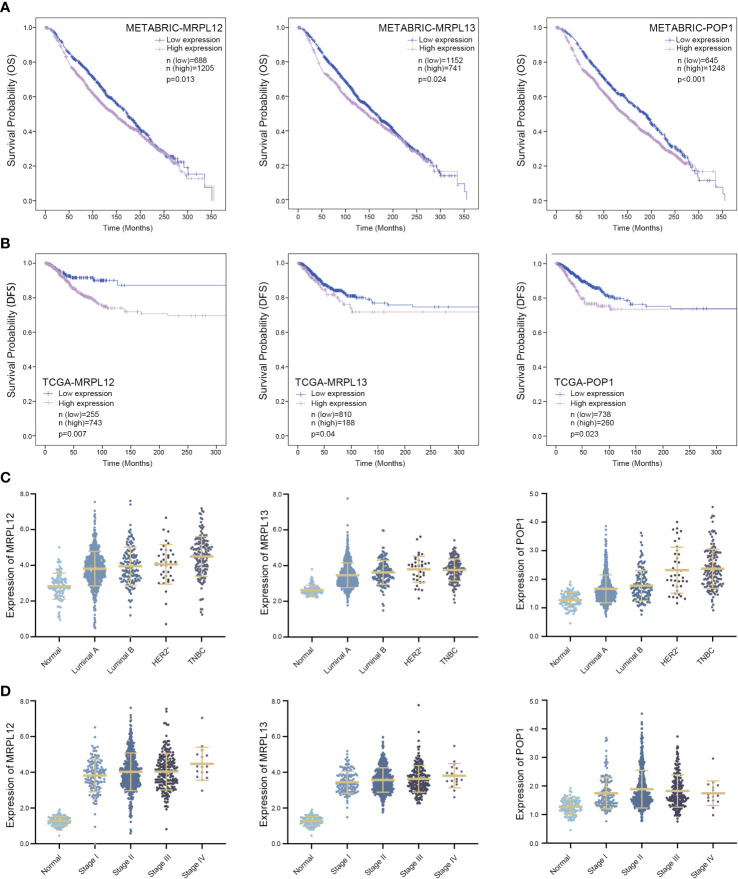
Three RBPs might be designed as prognostic biomarkers for breast cancer. **(A)** Survival Kaplan–Meier estimate of hub RBPs in the TCGA-BRCA database (n = 1,088), clinical samples were grouped by Youden index of mRNA expression level; **(B)** Kaplan–Meier plotter of these hub RBPs in the METABRIC database (n = 1,893), in which samples were grouped by Youden index of mRNA expression level; **(C)** Comparing the expression pattern of these hub RBPs in four subtypes of breast cancer versus normal tissue; **(D)** Correlation between the expression pattern of these hub RBPs and the tumor stage in patients suffering from breast cancer. DFS, disease-free survival; OS, overall survival and *p <* 0.05 was considered as statistically significant.

Take advantage of Lasso-penalized Cox analysis, 15 RBP-coding genes which might be prognosis signature for survival were screened from the aforementioned 62 RBPs (**Figure 2A**). Subsequently, 6 RBP-coding genes with *p*-value <0.05 were further identified as candidates by Stepwise-multivariate Cox regression analysis (**Supplementary Figures 1**, **2** and **Supplementary Table 4**). As shown in **Figure 4**, patients with highly expressed *MRPL12*, *MRPL13* or *POP1* always showed poorer disease-free survival (DFS, *p <*0.05) in the TCGA-BRCA dataset and poorer overall survival (OS, *p <*0.05) in the METABRIC dataset. Although *MRPL12*, *MRPL13* or *POP1* were over-expressed in tumor tissues, there were also some slight differences in expression pattern between each subtype of breast cancer, wherein TNBC cases tolerated the highest expression of these three RBPs (**Figure 4B** and **Supplementary Table 5**). In addition, the expression pattern of *MRPL12*, *MRPL13* or *POP1* were significantly associated with tumor stage (**Figure 4D** and **Supplementary Table 6**), suggesting that these three RBP-coding genes could be regarded as potential biomarkers for breast cancer prognosis.

**Figure 5:**
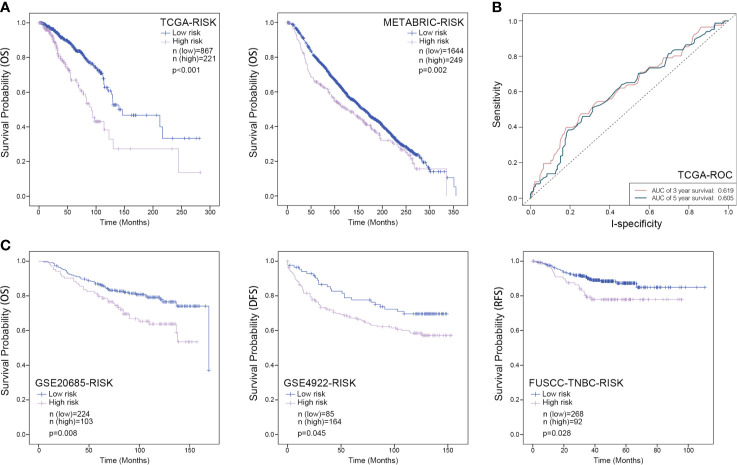
Kaplan–Meier analysis for the three-RBPs prognostic signature in breast cancer. **(A)** Survival Kaplan–Meier estimate of the three-RBPs prognostic signature in the TCGA-BRCA database (n = 1,088) and METABRIC database (n = 1,893), in which clinical samples were grouped by Youden index of risk score; **(B)**, ROC curve for assessing the reliability of the model; **(C)** Survival Kaplan–Meier estimate of the three-RBPs prognostic signature in the GSE20685 database (n = 327), the GES4922 database (n = 249) as well as the FUSCC database (n = 360), in which samples were grouped by Youden index of risk score. OS, overall survival; DFS, disease-free survival; RFS, recurrence-free survival. *P <*0.05 was considered as statistically significant.

Furthermore, a prognostic model based on these three RBP-coding genes was constructed by a linear combination of the regression coefficient and its mRNA expression level. In this model, the risk score of each patient was calculated by the equation that risk score = 0.282 ∗ Expression _MRPL13_ − 0.186 ∗ Expression _MPRL12_ + 0.288 ∗ Expression_POP1_. Patients were grouped into low- or high-risk set according to the Youden index of their risk score, and the Kaplan–Meier survival curve combined with a long-rank test was employed to assess whether these three RBP-coding genes can be used as a gene signature for predicting the survival probability of patients with breast cancer. Either within the TCGA-BRCA or METABRIC datasets, patients of high-risk were with poorer OS than those of low-risk (**Figure 5A**). However, the area under curve (AUC) value of ROC curves of this model was only 0.619 for predicting 3 years survival and 0.605 for predicting 5 years survival (**Figure 5B**). Moreover, the FUSCC-TNBC dataset (n = 360), GSE4922 cohort (n = 249) and GSE20685 cohort (n = 327) were introduced for external validation, which indicated a consistent result that patients in the high-risk group always showed significantly poorer survival probability than the low-risk group (**Figure 5C**), indicating a good performance of this three-gene signature for survival prediction of breast cancer patients.

### Building and Validation of a Predictive Nomogram

**Figure 6:**
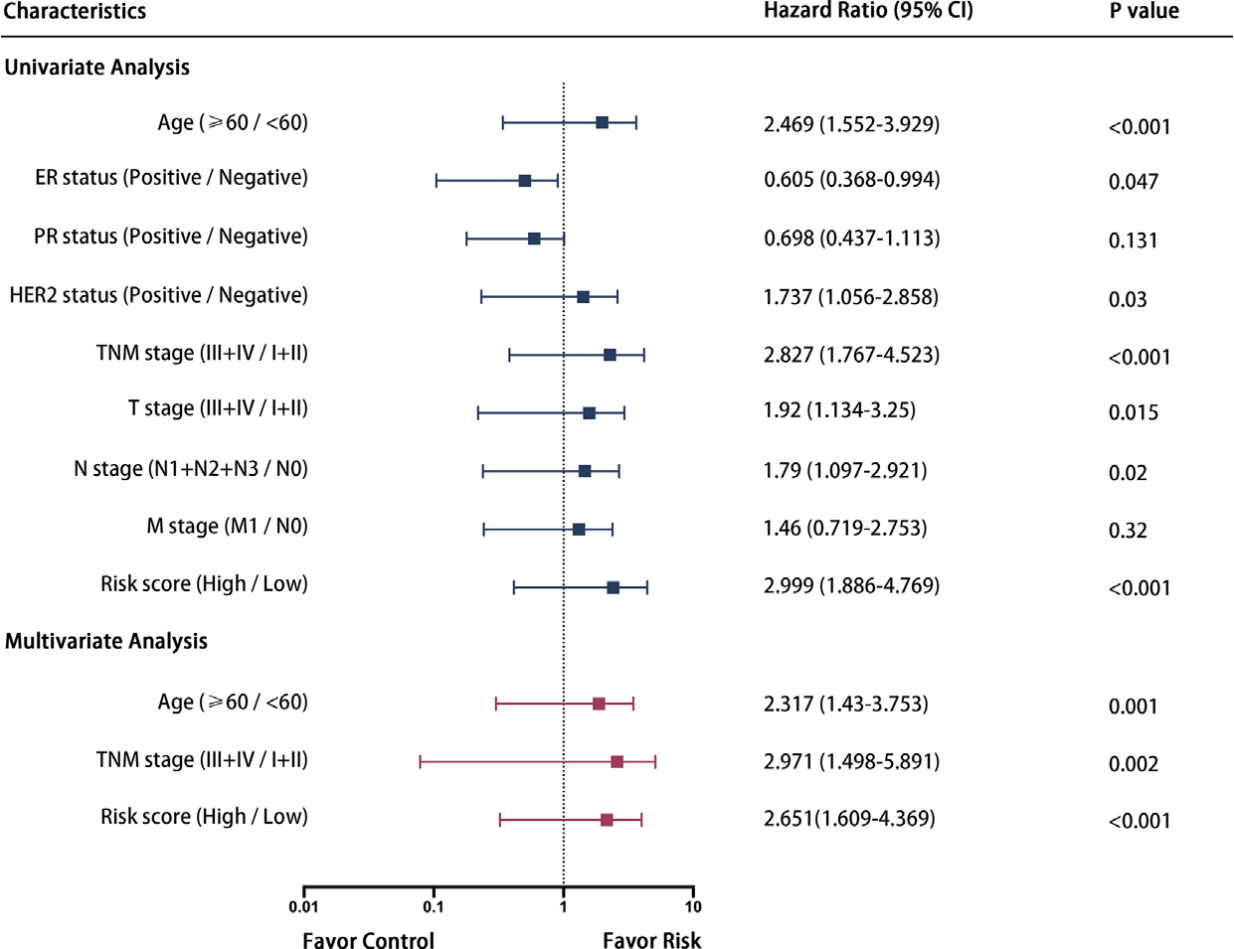
Forrest plot of the univariate and multivariate Cox regression analysis in breast cancer.

Some 687 patients from the TCGA-BRCA dataset with complete clinicopathological information including age, ER status, PR status, HER2 status and TMN stage were submitted for further analysis. As shown in **Figure 6**, univariate and multivariate Cox regression analysis indicated that age, TNM stage and the risk score calculated from the three-gene signature were independent prognostic factors for OS of breast cancer patients.

**Figure 7:**
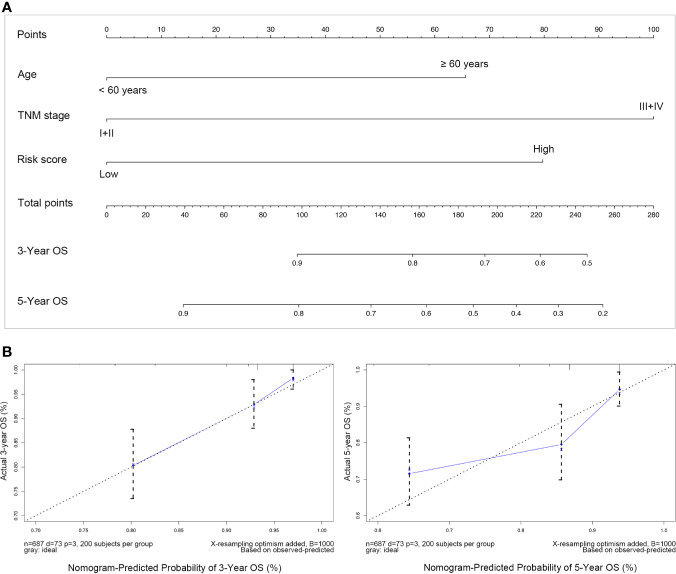
Nomogram predicting overall survival for breast cancer. **(A)** For each patient suffering from breast cancer, three lines should be drawn upward to determine the points received from the three predictors in the nomogram, then the sum of these points should be located on the “Total points” axis, following a line be drawn downward to determine the possibility of 3-years and 5-years overall survival; **(B)** Calibration plot for validating this nomogram, in which the Y-axis represented actual survival while the X-axis represented predicted survival by the above nomogram.

Subsequently, these three independent prognostic factors including age, TNM stage and risk score were used to build a nomogram to predict 3-year and 5-year OS of patients with breast cancer. As the calibration plots shown in **Figure 7**, this nomogram built with the combined model might under-estimate the mortality which would be the ideal nomogram for predicting short-term survival for patients with breast cancer.

### *MRPL12*, *MRPL13* and *POP1* Were Functioned as Oncogenes in Breast Cancer

**Figure 8:**
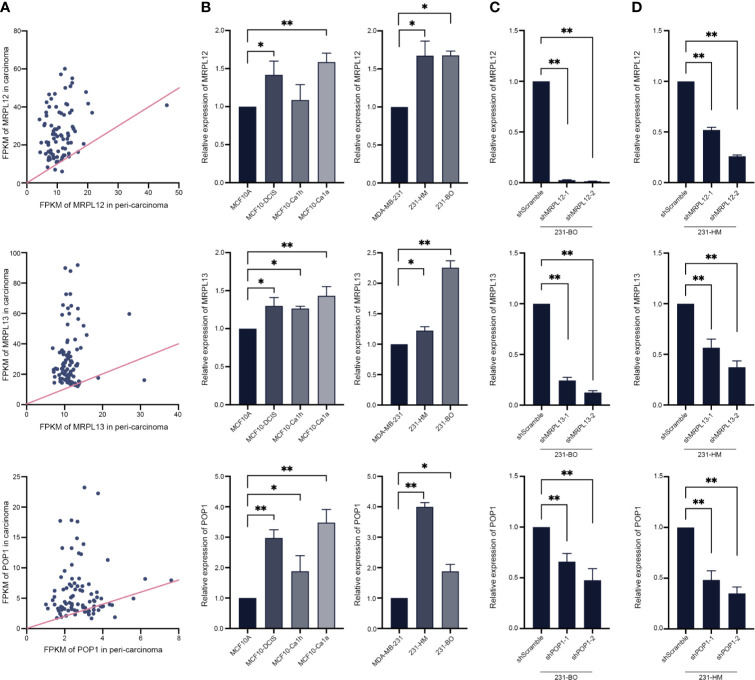
Experimental validation and perturbation of the expression pattern of hub RBPs in breast cancer cells. **(A)** The total RNA was isolated from 88 tumor-tissues and its para-carcinoma tissues from TNBC patient, and realtime-qPCR was carried out to compare the expression pattern of MRPL12, MRPL13 and POP1 between tumor and normal tissues. The straight line was an isoline, which means the equal expression pattern of hub genes in carcinoma and para-carcinoma tissues; **(B)** Realtime-PCR assay for evaluating the expression pattern of these three RBPs in a series of immortalized breast cancer cell lines (such as MDA-MB-231, bone metastatic 231-BO, lung metastatic 231-HM), the mammary epithelial cell line MCF10A as well as its derived pre-malignant cell line MCF10^DCIS^, malignant cell line MCF10^Ca1h^ and MCF10^Ca1a^; **(C, D)** Expression of the hub RBPs was down-regulated by RNA interference in 231-BO and 231-HM cells. *p < 0.05; **p < 0.01; Bars, ± SD.

In order to reveal the pathological function of these three RBPs in breast cancer, some routine experimental methods were employed. In line with the above statistical analysis, realtime-qPCR assay showed that either *MRPL12*, *MRPL13* or *POP1* were over-expressed in the clinical TNBC tissues rather than in the para-carcinoma tissues (**Figure 8A**). Similarly, *MRPL12*, *MRPL13* and *POP1* were also over-expressed in the pre-malignant cell line MCF10^DCIS^ as well as the malignant cell line MCF10^Ca1h^ and MCF10^Ca1a^ rather than in the mammary epithelial cell line MCF10A (**Figure 8B**). And the expression pattern of these three RBPs were more abundant in the metastatic 231-HM and 231-BO than in their parental MDA-MB-231 cells (**Figure 8B**). These results suggested that *MRPL12*, *MRPL13* and *POP1* might acted as oncogenes in breast cancer.

**Figure 9:**
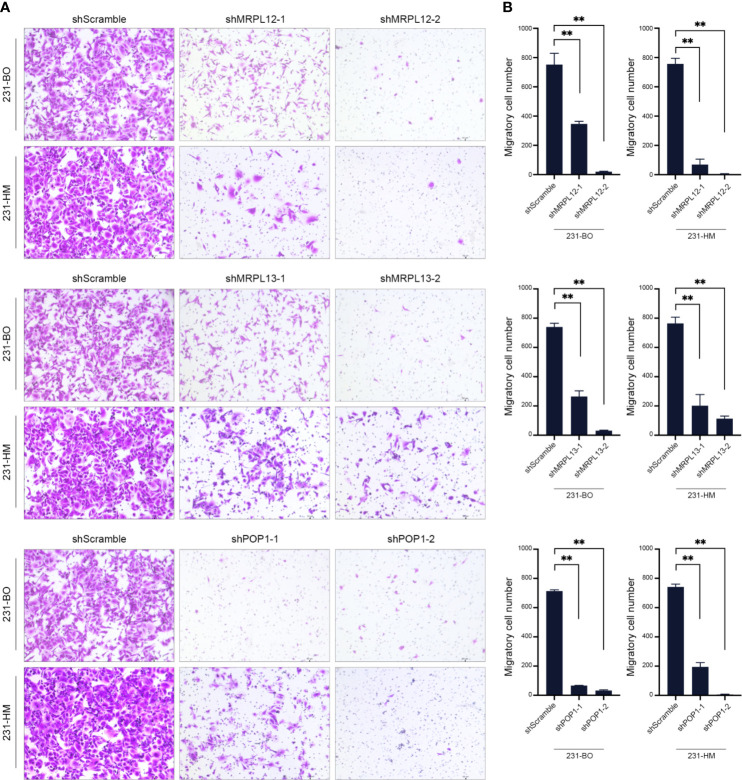
Influence of the hub RBPs on migration of breast cancer cells. **(A)** Transwell assay for evaluating the influence of down-regulating expression of the hub RBPs on migration of 231-BO and 231-HM cells; **(B)** Counting and statistical analysis of transwell assay. **p <0.01; Bars, ± SD.

**Figure 10:**
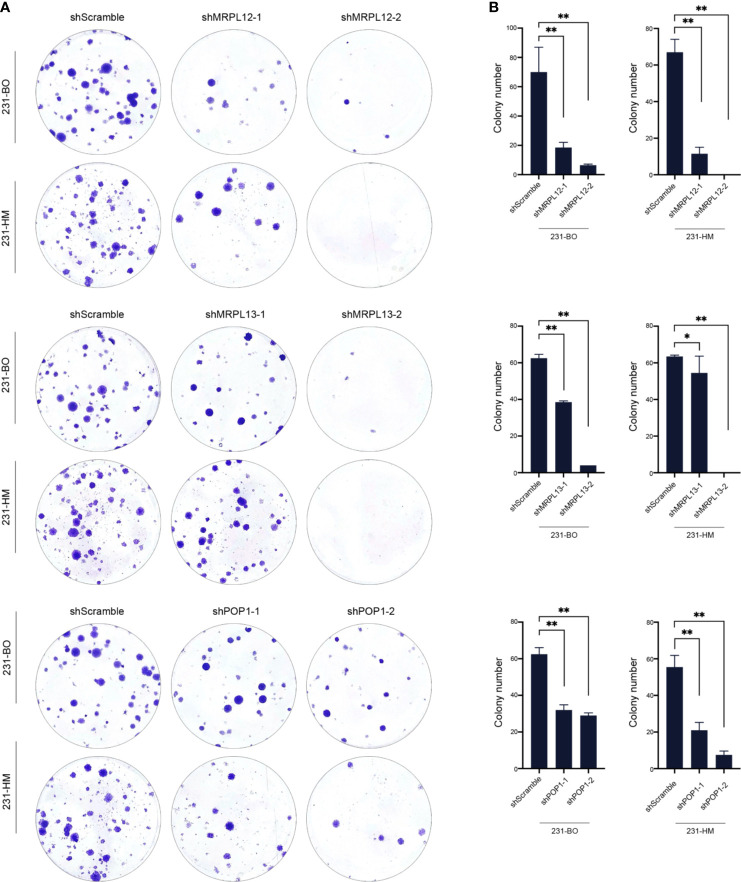
Influence of the hub RBPs on cellular viability of breast cancer. **(A)** Colony efficiency assay for evaluating the influence of down-regulating expression of the hub RBPs on cellular viability of 231-BO and 231-HM cells; **(B)** Counting and statistical analysis of colony efficiency assay. *p <0.05; **p <0.01; Bars, ± SD.

Furthermore, we down-regulating the expression of endogenous *MRPL12*, *MRPL13* or *POP1* in 231-HM and 231-BO cells using the specific shRNAs (**Figures 8C, D** **)**. Transwell assay indicated that down-regulating the expression of these three RBPs drastically decelerated the migration of breast cancer cells (**Figure 9**). Meanwhile, down-regulating the expression of these three RBPs also inhibited the cloning efficiency of breast cancer cells, suggesting that these three RBPs played an important role in maintaining the vitality of breast cancer cells (**Figure 10**). All these results confirmed that *MRPL12*, *MRPL13* and *POP1* acted as oncogenes in breast cancer cells, therefore, they might be regarded as prognostic indicators in breast cancer, but also could be designed as therapeutic targets of breast cancer.

### Target–Drug Interaction Network Analysis

**Figure 11:**
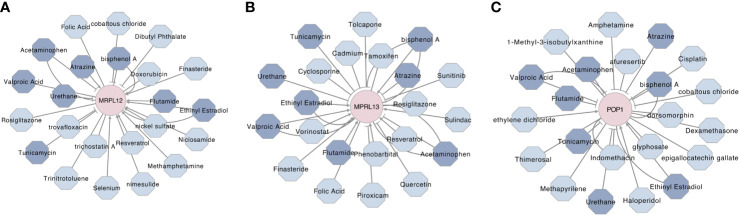
Prognostic biomarker-drug interaction network of the hub RBPs in breast cancer. The network illustrated some available chemicals that would increase or decrease the expression levels and/or activity of MRPL12 **(A)**, MRPL13 **(B)** and/or POP1 **(C)**. The triangle arrow presented up-regulation, while the vertical arrow presented down-regulation. Moreover, the chemicals which be highlighted by navy blue were predicted as the communal chemical that might influence these three hub RBPs, simultaneously.

To predict some available and potential compounds for targeting these three RBPs, a gene–compounds interaction network was constructed by using the Comparative Toxicogenomics Database (CTD) and *Cytoscape 3.8.0*. As shown in **Figure 11**, there were more than twenty compounds that could influence MRPL12, MRPL13 and/or POP1, wherein eight compounds could influence these three prognostic markers simultaneously. Thereinto, Ethinyl Estradiol could up-regulate the MRPL12, MRPL13 and POP1, while Acetaminophen, Bisphenol A, Urethane and Tunicamycin could negatively affect the physiological functions of these three RBPs. Flutamide and Valproic Acid might be used as agonist of MRPL12 and MRPL13, but might be used as antagonist against POP1. And Atrazine might be used as agonist of MRPL13 and POP1, but might be used as antagonist against MRPL12.

## Discussion

Currently, breast cancer remains the most prevalent malignancy in females, even though the advanced surgical and systemic treatments have successfully doubled the survival rate of patients (1). Breast cancer is characterized by multifactorial etiology and multigenetic alteration, which displays extraordinarily heterogeneity as well as capricious clinical phenotypes, confronts diverse clinical events and requires different therapeutic strategies (20). Therefore, it is vital to consider the subtypes of breast cancer as a prerequisite to any basic research and clinical regimens.

In this study, we introduced the prevailing four molecular subtypes of breast cancer to define the universality and specificity of the hub genes which would be designed as biomarkers in clinical practice of breast cancer. Generally, breast cancer is mainly classified into the Luminal-A (ER^+^/PR^high^/HER2^−^), Luminal-B (ER^+^/PR^low^/HER2^−^), HER2-riched (ER^−^/PR^-^/HER2^+^) and Basal-like (ER^−^/PR^−^/HER2^−^, also known as TNBC) subtypes according to the expression pattern of estrogen receptor (ER), progesterone receptor (PR) and human epidermal growth factor receptor 2 (HER2) (21, 22). As the retrial of TCGA-BRCA database, a total of 323 differential expressed RBP-coding genes were identified by comparing the transcriptome between tumor and normal tissues (**Supplementary Table 3**). However, only a fraction of these differently expressed RBPs were communal in four subtypes of breast cancer, while others showed specificity in one or more subtypes (**Figure 2**). Here, to develop a universal predictive model that would be rudely applied in early diagnosis and prognosis of breast cancer, we merely focused on the differential expressed RBP-coding genes which were overlapped between the four molecular subtypes of breast cancer, wherein 62 RBP-coding genes were qualified for further study.

To increase feasibility and accuracy of the prognostic model, a succession of screening was performed as depicted in the flow chart (**Figure 1**), which identified three RBP-coding genes (*MRPL12*, *MRPL13* and *POP1*) that possessed statistical significance in prognosis assessment of patients with breast cancer. No matter in the TCGA-BRCA dataset or in the METABRIC dataset, a significant negative-relationship always existed between the survival probability and the expression level of *MRPL12*, *MRPL13* or *POP1*, meaning that patients should undergo high risk in recurrence and deterioration of breast cancer if they were abundant in the expression of *MRPL12*, *MRPL13* and/or *POP1*. Naturally, these three RBP-coding genes were constructed into the prognostic model for breast cancer, in addition, the risk score of this model was an independent prognostic factor. As shown in **Figure 5**, this prognostic model worked well not only in the TCGA-BRCA and METABRIC datasets, but also be significant in the GSE20685, GSE4922 as well as the FUSCC-TNBC cohorts, in which patients in the high-risk group tended to undergo the more dismal survival probability than patients in the low-risk group. Moreover, a nomogram combining the 3-RBPs signature and conventional clinical parameters (such as age and TNM stage) performed the best in predicting survival probability for patients with breast cancer, indicating that this prognostic model developed from the three RBP-coding genes’ signature could be designed as a useful indicator for survival in breast cancer.

Consistent with statistical analysis of the public databases, we found that *MRPL12*, *MRPL13* and *POP1* were over-expressed in most of the TNBC-tissues than their para-carcinoma specimens, and be more abundant in the differentiated and metastatic breast cancer cell lines (such as MCF10^Ca1a^, 231-HM and 231-BO). Furthermore, down-regulating the expression of endogenous *MRPL12*, *MRPL13* or *POP1* in breast cancer cells, resulted in dramatically suppression of cellular viability and migration, suggesting that these three RBP-coding genes might act as oncogenes in accelerating the progress of breast cancer (**Figures 8**, **9**). All these results indicated that *MRPL12*, *MRPL13* and *POP1* not only could be designed as prognostic factors for predicting survival probability of patients with breast cancer, but also could be used as potential targets for clinical intervention of breast cancer.

As far as we know, MRPL12 and MRPL13 are components of the mitochondrial large ribosomal subunit, while POP1 has been identified as a communal component of the nuclear RNase P and the MRP ribonuclease complex (23–25). Mechanistically, MRPL12 and MRPL13 are major in maintaining the structural and functional integrity of mitoribosome, which is a dedicated apparatus for producing mtDNA-encoded multimeric oxidative phosphorylation enzymes and be essential in regulating the mitochondrial respiration and energy homeostasis (24, 26). Besides, MRPL12 also interacts directly with the mitochondrial RNA polymerase (POLRMT) and facilitates the mitochondrial transcription (27). Deficiency of either MRPL12 or MRPL13 can disrupt the mitoribosome and disturb the expression of mitochondrial genes, that eventually lead to loss of the functional mitochondrion (28). Accumulating evidence showed that the deformative and/or dysfunctional mitoribosome was responsible for some devastating malignancies, and a panel of mitoribosome components as well as its assembly factors could be regarded as hallmarks to plot the molecular portraits for specific tumors, such as MRPS23 be significantly in the breast cancer, while DDX28 acts as a risk factor for colorectal cancer (29, 30). Here, we had identified that MRPL12 and MRPL13 also could be regarded as potential risk factors for breast cancer. In this context, some strategies that targeting the MRPL12, MRPL13 or even the mitoribosome might be practicable in curing breast cancer.

Moreover, analogous to MRPL12 and MRPL13, our results also revealed that POP1 functioned as an oncogene in breast cancer. In brief, majority of patients with abundant POP1 expression showed poorer survival probability, and silencing the endogenous POP1 dramatically suppressed the viability and metastatic ability of breast cancer cells (**Figures 4**, **9**, **10**). Previous studies have reported that POP1 plays the role of a scaffold for stabilizing the global architecture of RNase P and MRP, which are primarily responsible for the maturation and metabolism of tRNA, rRNA as well as some mRNA (25, 31, 32). Biallelic mutation or haploinsufficiency of POP1 impairs the holoenzyme assembly and results in a severe skeletal dysplasia, but whether it is involved in tumorigenesis still is unsubstantiated (33).

Notably, by reviewing the Comparative Toxicogenomics Database (CTD), we excavated three available compounds (Acetaminophen, Urethane and Tunicamycin), which could inhibit the MRPL12, MRPL13 and POP1 simultaneously, implying their anti-neoplastic effects on breast cancer (**Figure 11**). Coinciding with our prediction, some studies have reported that Acetaminophen, one of the most widely used anti-inflammatory, antipyretic and analgesic drug, could inhibit the tumor growth by inducing the differentiation of breast cancer stem cells (34). Tunicamycin could induce apoptosis, decelerate growth and aggressiveness of breast cancer cells *via* the Akt/NF-κB signaling pathway (35). Meanwhile, Tunicamycin could enhance the anti-neoplastic activity of trastuzumab on breast cancer (36), suggesting a possibility that these compounds should be applied into the systemic therapeutic strategies for breast cancer.

## Conclusion

Oval all, we identified three RBP-coding genes (*MRPL12*, *MRPL13* and *POP1*) that functioned as risk factors and independent prognostic factors for breast cancer. Naturally, a three-RBPs-signature based prognostic model was established, and be validated for predicting the survival probability of patients with breast cancer. Furthermore, we performed some preliminary experiments and provided concrete evidence that *MRPL12*, *MRPL13* and *POP1* might be oncogenes in maintaining cellular viability as well as accelerating metastasis of the breast cancer cells. All these results suggested that MRPL12, MRPL13 and POP1 should be designed as biomarkers and potential intervening targets for breast cancer. However, more in-depth studies are required to reveal the specific molecular mechanisms and to verify the related clinical practices.

## Data Availability Statement

The original contributions presented in the study are included in the article/**Supplementary Material**. Further inquiries can be directed to the corresponding authors.

## Ethics Statement

The studies involving human participants were reviewed and approved by Ethics Committee of Fudan University Shanghai Cancer Center. The patients/participants provided their written informed consent to participate in this study.

## Author Contributions

YL and HS conceived of and designed the study. QL, YZ, LL, and BX contributed to the literature search. YL and XL performed the data analysis. YL wrote the initial draft of this manuscript. YH and WJ reviewed and edited the manuscript. All authors contributed to the article and approved the submitted version.

## Funding

This work was supported by the grants from National Natural Science Foundation of China (81972727 and 82002796).

## Conflict of Interest

The authors declare that the research was conducted in the absence of any commercial or financial relationships that could be construed as a potential conflict of interest.

## Supplementary Material

Supplementary Figure 1 Lasso-penalized Cox analysis. **(A)** Lasso coefficient profiles; **(B)** Cross-validation for tuning parameter selection in the Lasso model.

Supplementary Figure 2 Kaplan-Meier analysis for the prognostic significant of other RBPs in breast cancer. **(A, B)** Survival Kaplan-Meier estimate of RBPs identified by stepwise-multivariate Cox regression analysis in the TCGA-BRCA database (n=1088), clinical samples were grouped by Youden index of mRNA expression level; B, Kaplan-Meier plotter of EFI4E3 or NOVA2 in the METABRIC database (n=1893), in which samples were grouped by Youden index of mRNA expression level. OS, overall survival and *p*<0.05 was considered as statistically significant.
