# Supplementary figures and images for: Identification of a Three-RNA Binding Proteins (RBPs) Signature Predicting Prognosis for Breast Cancer
Source: Front Oncol. 2021 Jul 12;11:663556. doi: 10.3389/fonc.2021.663556 (PMC8311660; doi:10.3389/fonc.2021.663556)

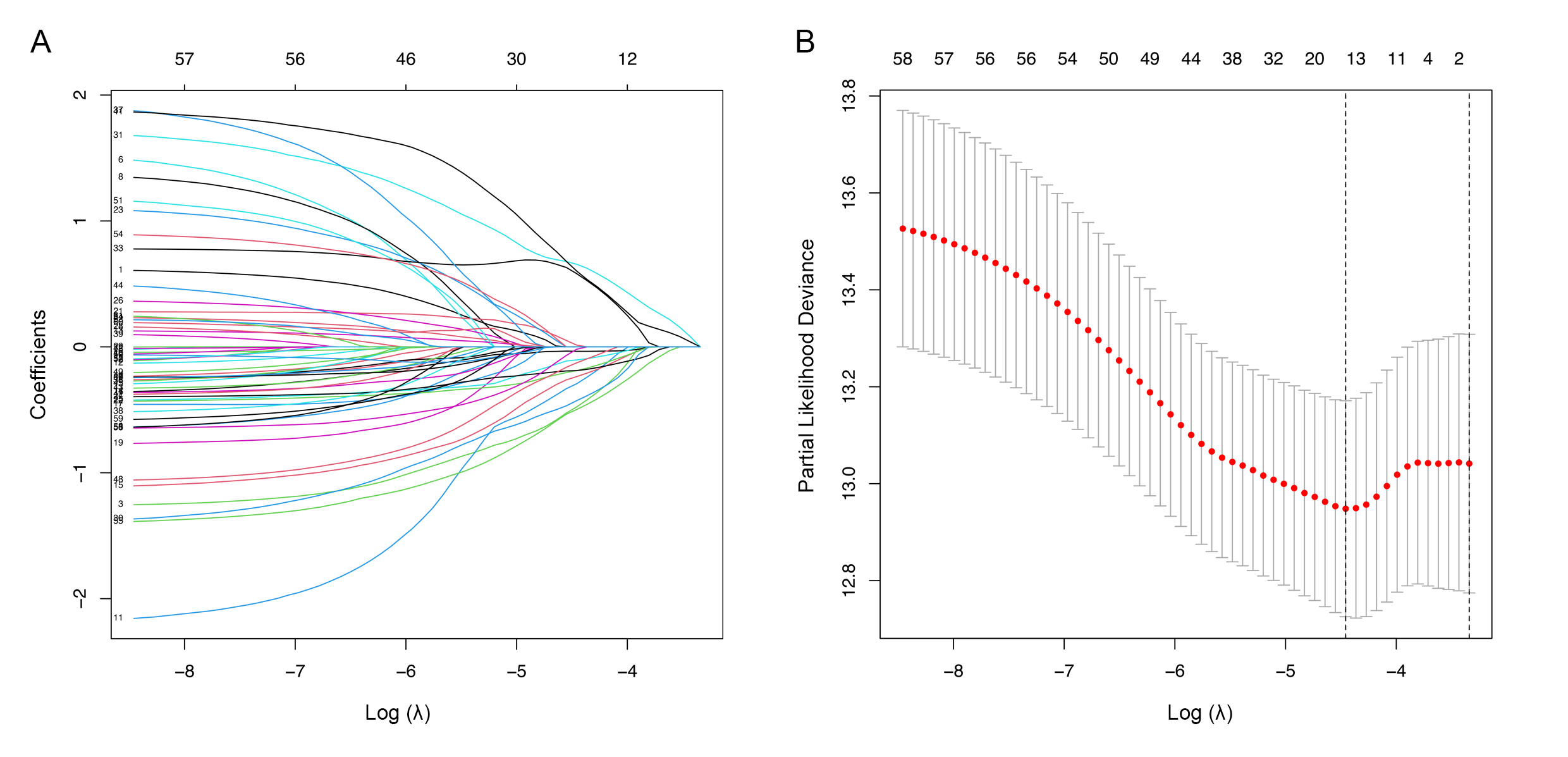

Supplement: Supplementary Figure 1 — Lasso-penalized Cox analysis. (A) Lasso coefficient profiles; (B) Cross-validation for tuning parameter selection in the Lasso model. [file Image_1.tif]

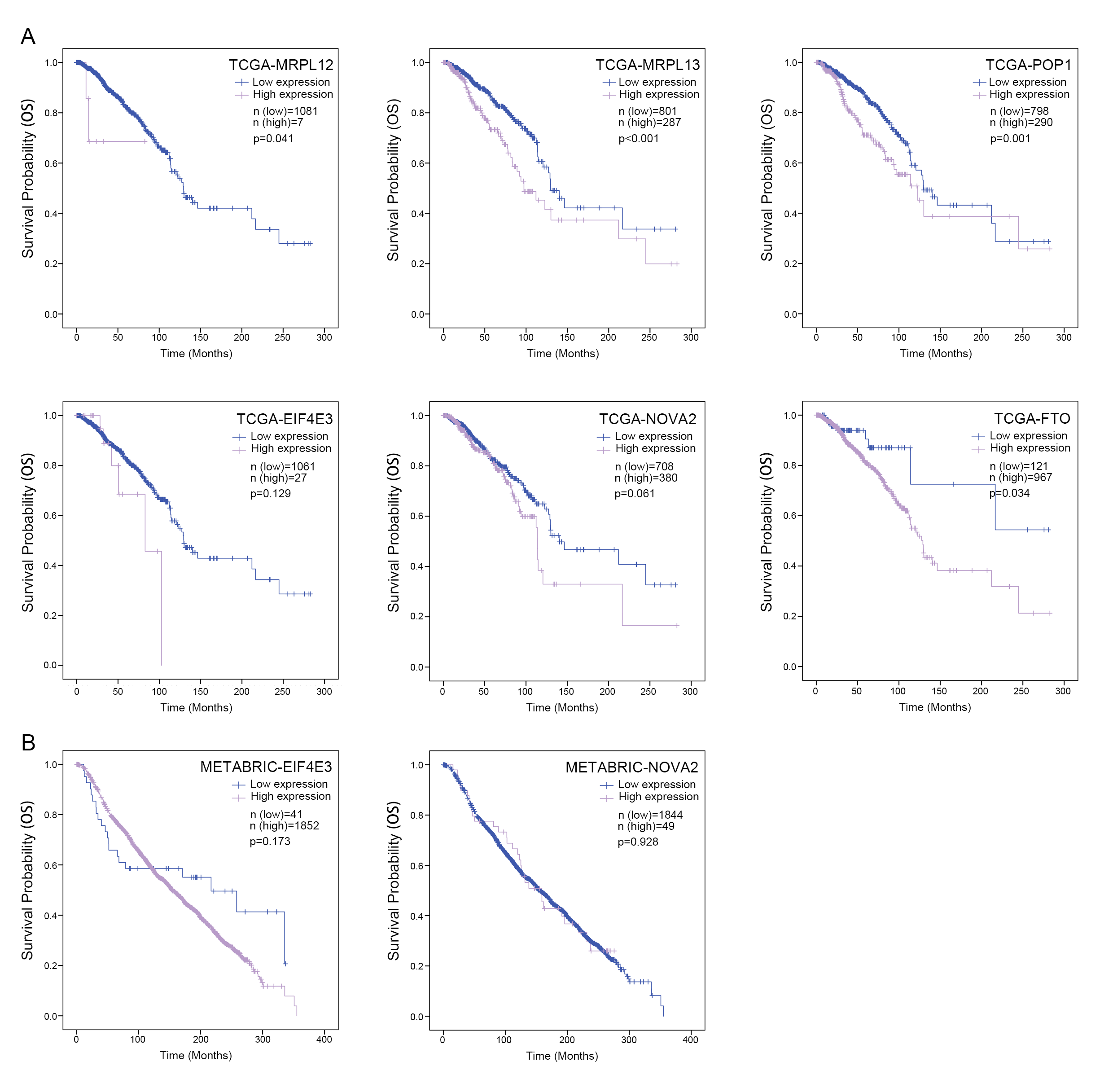

Supplement: Supplementary Figure 2 — Kaplan-Meier analysis for the prognostic significant of other RBPs in breast cancer. (A, B) Survival Kaplan-Meier estimate of RBPs identified by stepwise-multivariate Cox regression analysis in the TCGA-BRCA database (n=1088), clinical samples were grouped by Youden index of mRNA expression level; B, Kaplan-Meier plotter of EFI4E3 or NOVA2 in the METABRIC database (n=1893), in which samples were grouped by Youden index of mRNA expression level. OS, overall survival and p<0.05 was considered as statistically significant. [file Image_2.tif]
